# Comparative Multi-Omics Analysis of Rhizome Shooting in *Fargesia rufa* Under Altitudinal Temperature Variation

**DOI:** 10.3390/plants15152302

**Published:** 2026-07-27

**Authors:** Xin Zhao, Man Tang, Yanwen Zhao, Mengqiu Chen, Xiaojun Wang, Qi Lin, Zhijian Long, Shanglian Hu

**Affiliations:** 1 College of Life Science and Agri-Forestry, Southwest University of Science and Technology, Mianyang 621000, China; 2 Sichuan Provincial Forestry and Grass Land Key Laboratory for Conservation and Sustainable Utilization of Bamboo Genetic Resources in Southwest of China, Mianyang 621010, China; 3 Key Laboratory of Bamboo Cultivation and Management, National Forestry and Grassland Administration, Mianyang 621000, China; 4 The Conservation of Endangered Wildlife Key Laboratory of Sichuan Province, Sichuan Academy of Giant Panda, Chengdu 610000, China

**Keywords:** *Fargesia rufa*, rhizome shooting, altitudinal gradient, soil temperature, integrated metabolomics and transcriptomics

## Abstract

Bamboo shoots, as the most nutritionally valuable food source for giant pandas during key reproductive seasons, are critical for conservation because their availability and timing directly influence panda foraging and habitat use. However, the molecular mechanisms through which altitudinal temperature variation governs rhizome shooting in staple food bamboos remain largely unknown. Here, we performed integrated metabolomic and transcriptomic analyses of *Fargesia rufa* rhizomes collected along an elevational gradient (1000 m, 1500 m, and 2000 m), with a critical paired comparison at 2000 m between a non-shooting cold gully-edge site (16.4 °C) and a shooting warm gully-center site (21.1 °C), where soil temperature is elevated by approximately 4 °C due to prolonged solar exposure. Our results demonstrate that soil temperature, rather than elevation per se, acts as the primary driver of rhizome shooting, with an apparent threshold near 20 °C. A core shooting metabolome (CSM) comprising 843 metabolites was consistently accumulated across all shooting conditions, which featured gibberellin/auxin precursors, TCA cycle intermediates, and phenylpropanoid compounds. Correspondingly, a core shooting transcriptome (Rh_shooting) of 10,970 genes was identified, which resolved into three functionally distinct temporal clusters: “shooting-on” (activated upon threshold crossing, enriched in hormone signaling and cell wall metabolism), “temperature-dose” (progressively upregulated with rising temperature, enriched in energy metabolism and defense), and “microenvironment-enhanced” (specifically upregulated in the high-elevation warm gully, enriched in photosynthesis and antioxidant pathways). Integrative network analysis further revealed zeatin riboside and multiple hub genes as central coordinators linking hormone signaling, energy metabolism, and cell wall remodeling. Collectively, these findings establish a molecular framework linking altitudinal temperature variation to bamboo rhizome regeneration—a process that directly determines the spatiotemporal availability of bamboo shoots for giant pandas. This work provides mechanistic insights into giant panda foraging ecology and has direct implications for predicting habitat quality under climate change and informing evidence-based conservation strategies for this flagship species and its critical food resource.

## 1. Introduction

*Fargesia rufa* is one of the principal food bamboos for the giant panda and is extensively distributed across the mountainous regions of southern Gansu and northern Sichuan, typically occurring at elevations of 1000–2000 m [1]. As a typical clonal species with a shallow root system, *F. rufa* is particularly sensitive to environmental variables including soil moisture, temperature, and light availability. Among the various environmental factors influencing its growth and regeneration, temperature has been recognized as a primary trigger for bamboo shooting [2,3]. In natural populations, *F. rufa* exhibits a striking altitudinal gradient in its shooting phenology: shoot emergence progresses sequentially from lower to higher elevations, with marked altitudinal differences in shoot growth rates, lignification timing, and nutrient accumulation patterns [4,5]. This gradient integrates declining temperature, intensified UV, reduced atmospheric pressure, and increased diurnal temperature fluctuations, offering a natural system to investigate the molecular basis of bamboo adaptation to altitude [1,6,7].

The giant panda (*Ailuropoda melanoleuca*) has evolved an obligate bamboo-based diet, making its population viability critically dependent on bamboo resource dynamics [8,9]. Among its foraging strategies, the “shoot-chasing” phenomenon—where pandas migrate upward to track the sequential emergence of bamboo shoots along the altitudinal gradient as temperatures rise in spring and summer—directly links bamboo shoot availability to panda movement patterns and habitat use [10,11,12]. However, while the ecological correlates of this panda–bamboo interaction have been well documented [13,14,15], the molecular mechanisms within the bamboo rhizome that govern altitudinal variation in shooting remain largely unexplored.

The rhizome—the underground stem of bamboo—serves as the primary storage organ responsible for bud dormancy and germination [16,17]. Rhizome buds develop into bamboo shoots, and the shooting process is directly governed by the physiological and molecular status of the rhizome. Environmental modulation of bamboo metabolism and development is intimately linked to transcriptional regulation [18]. In various bamboo species, rhizome bud germination has been shown to be cooperatively regulated by multiple pathways including carbohydrate metabolism, hormone signal transduction, cell wall biogenesis, and temperature response [19,20]. Endogenous phytohormones such as indole-3-acetic acid (IAA), zeatin riboside (ZR), gibberellin (GA), and abscisic acid (ABA) play central roles in regulating the transition of rhizome buds from dormancy to germination [21,22]. Temperature signals are perceived and transduced through complex molecular networks that ultimately modulate gene expression and metabolic flux [23,24,25]. However, most studies on altitudinal adaptation have focused on leaves or aboveground organs [26], the rhizome—which directly controls shooting and thus shoot availability for pandas—has received far less attention. Consequently, the regulatory relationships linking soil temperature, gene expression, and metabolite synthesis in *F. rufa* rhizomes across altitudes remain largely unclear. There is a critical need for integrated multi-omics analyses that systematically characterize the molecular reprogramming of rhizomes in response to temperature variation along altitudinal gradients as this knowledge is essential for understanding the regeneration dynamics of this ecologically critical bamboo species and, by extension, the foraging ecology of giant pandas.

Therefore, we conducted an integrated metabolomic and transcriptomic analysis of *F. rufa* rhizomes collected from three elevations (1000 m, 1500 m, and 2000 m), with a critical paired comparison at 2000 m: a non-shooting population at the cold gully edge (16.4 °C) versus a shooting population in the warm gully center (21.1 °C), where soil temperature is elevated by approximately 4 °C due to prolonged solar exposure. This design allows us to disentangle the effects of elevation from those of temperature. We aimed to: (i) identify the environmental factors most strongly associated with rhizome shooting; (ii) characterize the metabolic and transcriptional signatures distinguishing shooting from non-shooting rhizomes; (iii) define the core metabolite and gene sets constituting the molecular machinery of shooting; and (iv) construct an integrative regulatory network linking environmental signals to downstream molecular responses. Elucidating these molecular mechanisms is essential for understanding bamboo regeneration dynamics and, by extension, the foraging ecology of giant pandas, with direct implications for habitat conservation under climate change.

## 2. Results

### 2.1. Analysis of Soil Physicochemical Properties in F. rufa Growing Areas at Different Elevations

**Figure 1:**
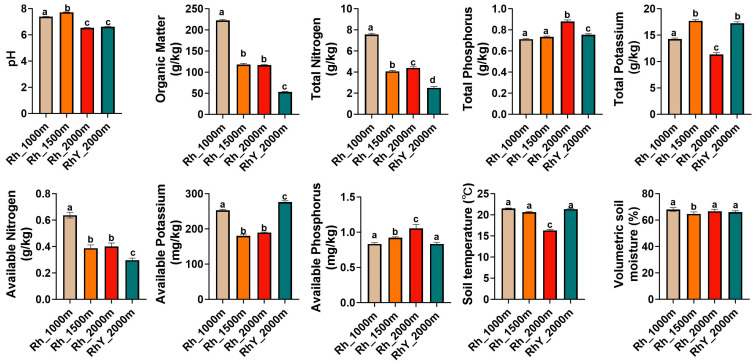
Analysis of soil physicochemical properties in *F. rufa* growing areas at different elevations. Soil physicochemical properties of *F. rufa* at four sites across three different elevations, including pH, organic matter, total nitrogen, total phosphorus, total potassium, available nitrogen, available potassium, available phosphorus, and soil temperature, volumetric soil moisture. The sampling sites are denoted as Rh_1000m (1000 m elevation, gully edge, shooting), Rh_1500m (1500 m elevation, gully edge, shooting), RhY_2000m (2000 m elevation, gully center (valley), shooting), and Rh_2000m (2000 m elevation, gully edge, non-shooting). Different lowercase letters indicate significant differences among samples.

To elucidate the environmental factors underlying altitudinal variation in rhizome shooting of *F. rufa*, we collected rhizomes and corresponding rhizosphere soils from shooting populations at 1000 m (Rh_1000m, gully edge), 1500 m (Rh_1500m, gully edge), and 2000 m (RhY_2000m, gully center, where “Y” denotes valley), alongside a non-shooting population at the gully edge of the same elevation (Rh_2000m), all situated within a single valley system. Thus, the prefix “Y” in RhY_2000m distinguishes the gully-center (valley) microhabitat from the gully-edge sites (Rh_1000m, Rh_1500m, and Rh_2000m). Soil physicochemical analyses (Figure 1) revealed that soil temperature declined progressively with increasing elevation along the gully-edge transect (1000 → 1500 → 2000 m), from approximately 22 °C to 16 °C. In contrast, total nitrogen, total phosphorus, total potassium, volumetric soil moisture and pH did not exhibit any consistent monotonic trends across the same gradient. At 2000 m, the gully-center shooting site registered a markedly higher soil temperature (21.1 °C) than the adjacent gully-edge non-shooting site (16.4 °C), representing a difference of approximately 4 °C—a disparity attributable to the central-slope position receiving substantially prolonged solar radiation. Notably, the soil temperature at the 2000 m shooting site (21.1 °C) was comparable to that at the 1000 m shooting site (21.6 °C), and both significantly exceeded the temperature at the 2000 m non-shooting site (16.4 °C). Collectively, these results indicate that soil temperature is the environmental factor most strongly associated with rhizome shooting status across the studied elevation gradient.

### 2.2. Association Between Soil Factors and Multi-Omics Data Revealed by RDA

**Figure 2:**
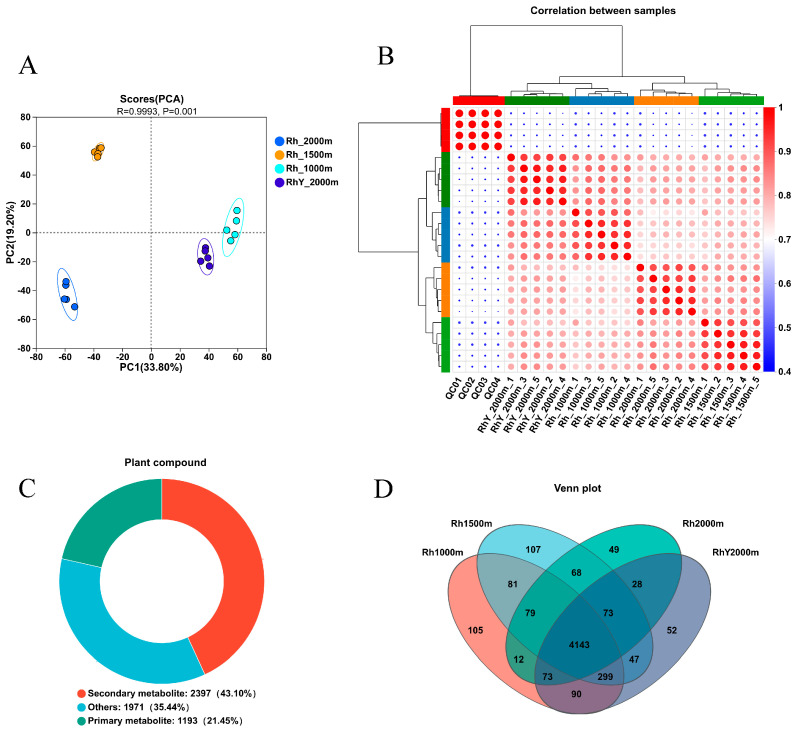
Metabolomic analysis of *F. rufa* rhizomes at different elevations. (**A**) PCA score plot. Samples were analyzed by dimensionality reduction, and the relative coordinates of each sample are shown on principal components P1 and P2. The distance between coordinate points represents the degree of aggregation or dispersion among samples; closer distances indicate higher similarity, while greater distances indicate larger differences. (**B**) Correlation heatmap. The bottom of the figure shows the sample names of this project. The left and top sides display the clustering of each sample. Colored blocks represent the correlation between two samples; higher values indicate stronger correlation and greater similarity, reflecting better biological replicates among samples. QC (quality control) samples (labeled as OC01−04 in the figure) were prepared by pooling equal volumes of all sample extracts and were injected periodically throughout the analytical sequence to monitor instrument stability and data reproducibility. The tight clustering of QC samples confirms high data quality and instrumental reproducibility. (**C**) Compound classification statistics. Based on the selected classifications (plant compound classification, plant primary metabolite classification, and plant secondary metabolite classification), the number of metabolites in each category is displayed in descending order, showing the characteristics and percentage of metabolites in each classification. Different colors in each pie chart represent different classifications, and the area indicates the relative proportion of metabolites in that category. (**D**) Venn diagram analysis. Different colors represent different groups (or samples). The numbers in the overlapping regions indicate metabolites shared among multiple groups, while the numbers in non–overlapping regions indicate metabolites unique to the corresponding group.

To quantitatively evaluate the contributions of soil physicochemical factors to metabolomic and transcriptomic variations, we performed redundancy analysis (RDA) using soil temperature, pH, total nitrogen, total phosphorus, total potassium, organic matter, available nitrogen, available potassium, available phosphorus, and soil moisture as explanatory variables (Supplementary Figure S2). At the metabolomic level (Supplementary Figure S2A), the RDA1 and RDA2 axes cumulatively explained 55.91% of the total variation. The soil temperature vector pointed toward the upper-left quadrant, showing a close positive association with the Rh_1000m (21.6 °C) and RhY_2000m (central gully, 21.1 °C) samples positioned in this region, indicating that favorable thermal conditions promoted the accumulation of shooting-associated metabolites. At the transcriptomic level (Supplementary Figure S2B), the RDA1 axis alone explained 80.01% of the variation, reflecting the high sensitivity of gene expression to soil environmental gradients. Although the temperature vector in Figure 2B was somewhat deflected due to multicollinearity among the multiple explanatory variables, combined with field observations, the Rh_2000m (gully edge, 16.4 °C) samples—which failed to produce shoots under low temperature stress—exhibited clear spatial separation from all shooting groups (Rh_1000m, Rh_1500m, and RhY_2000m) in the ordination space.

Collectively, these RDA results demonstrate that soil temperature not only serves as the primary factor differentiating the molecular profiles between low- and high-elevation samples, but also operates through an apparent physiological threshold near 20 °C. When soil temperature exceeds this threshold (e.g., at 1000 m and the central gully at 2000 m), it induces gene expression patterns and metabolic fluxes conducive to rhizome shooting; conversely, when temperature falls below this threshold (e.g., at the gully edge of 2000 m), these physiological processes are suppressed, leading to shooting failure. These findings, from a multi-dimensional “gene–metabolism–environment” perspective, confirm that soil temperature is the dominant factor limiting rhizome shooting at high elevations.

### 2.3. Metabolomic Profiling of F. rufa Rhizomes Across Different Altitudes

To characterize the metabolic landscape associated with altitudinal variation in rhizome shooting, we performed untargeted metabolomic analysis on rhizomes collected from 1000 m (shooting, Rh_1000m), 1500 m (shooting, Rh_1500m), and 2000 m elevations—specifically, a non-shooting population at the gully edge (Rh_2000m) and a shooting population in the gully center (RhY_2000m). As the primary storage organ governing bamboo shoot bud germination (Supplementary Figure S1), the metabolic status of rhizomes directly determines shooting competence. Metabolite profiling was conducted using liquid chromatography-mass spectrometry (LC-MS). Principal component analysis (PCA) resolved all samples into four distinct clusters (Figure 2A). Notably, Rh_1000m and RhY_2000m were positioned in close proximity within the PCA space, whereas Rh_1500m and Rh_2000m occupied separate regions. Of particular note, the two 2000 m populations—Rh_2000m (non-shooting) and RhY_2000m (shooting)—exhibited substantial separation along the principal components, indicating that an approximately 4 °C difference in soil temperature drives profound metabolic reprogramming. The PCA model parameters (R = 0.9993, *p* = 0.001) demonstrated excellent fit and validated the reliability of the metabolomic dataset. Pairwise sample correlation analysis further confirmed high reproducibility among biological replicates and pronounced metabolic divergence among elevation–microhabitat groups (Figure 2B).

Across all samples, a total of 2397 annotated secondary metabolites, 1193 primary metabolites, and 1971 other metabolites were identified (Figure 2C). Among secondary metabolites, flavonoids and terpenoids were the most abundant classes, each comprising 730 compounds. Within primary metabolites, amino acids and their derivatives accounted for 279 compounds, carbohydrates and their derivatives for 334, and lipids for 501 (Supplementary Figure S3). Venn diagram analysis revealed that 4143 metabolites were commonly present across all groups (core metabolome), alongside numerous elevation- and microenvironment-specific metabolites. For example, 105 metabolites were unique to Rh_1000m, whereas only 49 metabolites were specific to Rh_2000m (Figure 2D). Collectively, these results demonstrate that both elevation and microenvironmental temperature differences profoundly shape the metabolomic composition of *F. rufa* rhizomes. Moreover, elevated temperature at the same elevation (RhY_2000m) drove metabolic convergence toward the low-elevation shooting group (Rh_1000m), while cooler conditions (Rh_2000m) yielded a metabolically distinct profile, underscoring temperature as a key determinant of rhizome metabolic reprogramming.

**Figure 3:**
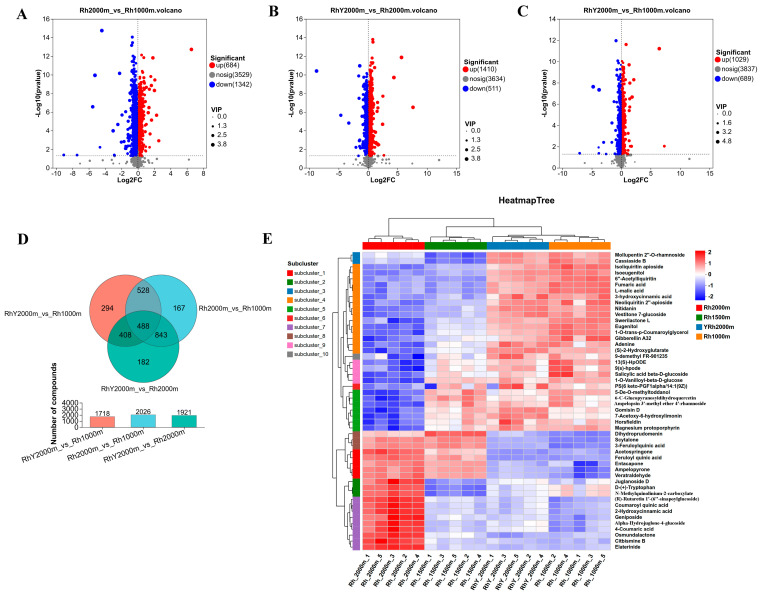
Differential metabolite analysis of *F. rufa* rhizomes across different elevations. (**A**) Volcano plot of differentially accumulated metabolites (DAMs) in *F. rufa* rhizomes between the 2000 m non-shooting site and the 1000 m shooting site. (**B**) Volcano plot of DAMs in *F. rufa* rhizomes between the 2000 m shooting site and the 2000 m non-shooting site. (**C**) Volcano plot of DAMs in *F. rufa* rhizomes between the 2000 m shooting site and the 1000 m shooting site. (**D**) Venn diagram illustrating the overlap of DAMs in *F. rufa* rhizomes across different elevations (shooting vs. non-shooting). (**E**) Clustering analysis of DAM sets in *F. rufa* rhizomes across different elevations (shooting vs. non-shooting). The heatmap displays the top 50 metabolites from the CSM (Core Shooting Metabolome), which were selected as those commonly differentially accumulated in both shooting vs. non-shooting comparisons [(Rh_1000m vs. Rh_2000m) and (RhY_2000m vs. Rh_2000m)] while excluding metabolites that were also differentially accumulated between the two shooting groups (Rh_1000m vs. RhY_2000m).

To pinpoint metabolites directly associated with the shooting phenotype while minimizing confounding effects of elevation, microhabitat, and temperature, we performed two independent pairwise contrasts: (i) Rh_1000m (shooting) versus Rh_2000m (non-shooting), representing the elevational gradient; and (ii) RhY_2000m (shooting, gully center) versus Rh_2000m (non-shooting, gully edge), representing the temperature-driven contrast at the same elevation. Differentially accumulated metabolites (DAMs) were defined using thresholds of VIP > 1.0, *p* < 0.05, and absolute fold change (|FC|) > 2. Volcano plots revealed substantial bidirectional abundance shifts across all comparisons. In the elevational contrast (Rh_2000m vs. Rh_1000m; Figure 3A), 684 metabolites were upregulated and 1342 were downregulated. In the RhY_2000m vs. Rh_1000m comparison (similar temperature but different elevations; Figure 3C), 1029 metabolites increased and 689 decreased. Notably, the temperature-driven comparison (RhY_2000m vs. Rh_2000m; Figure 3B) exhibited the most extensive metabolic reprogramming, with 1410 upregulated and 511 downregulated metabolites. Venn diagram analysis (Figure 3D) was employed to refine the DAM sets. By intersecting the two “shooting vs. non-shooting” contrasts (Rh_2000m vs. Rh_1000m and RhY_2000m vs. Rh_2000m) and excluding metabolites present in the RhY_2000m vs. Rh_1000m comparison (shooting vs. shooting), we obtained a refined set of 843 metabolites most directly associated with the shooting phenotype. This set was designated as the Core Shooting Metabolome (CSM), which represents the metabolic signature most directly linked to rhizome shooting, independent of elevation or microhabitat variation. These metabolites are robust against confounding from elevation or microhabitat differences and serve as potential metabolic biomarkers for rhizome shooting.

Hierarchical clustering of the CSM revealed distinct co-regulated modules. The heatmap of the top 50 most significantly altered metabolites (Figure 3E) highlighted several functionally relevant compounds, including the gibberellin precursor gibberellin A32, the auxin (IAA) biosynthetic precursor D-(+)-tryptophan, the phenylpropanoid intermediate 4-coumaric acid, and the phenolic signaling molecules acetosyringone and feruloyl quinic acid. The accumulation patterns of these metabolites align closely with the physiological demands of shoot germination, encompassing enhanced energy metabolism (TCA cycle), activated hormone biosynthesis (GA and IAA precursor accumulation), cell wall remodeling (phenylpropanoid metabolism), and initiation of antioxidant defense.

### 2.4. KEGG Pathway Enrichment and Key Metabolite Expression Profiling of the CSM

**Figure 4:**
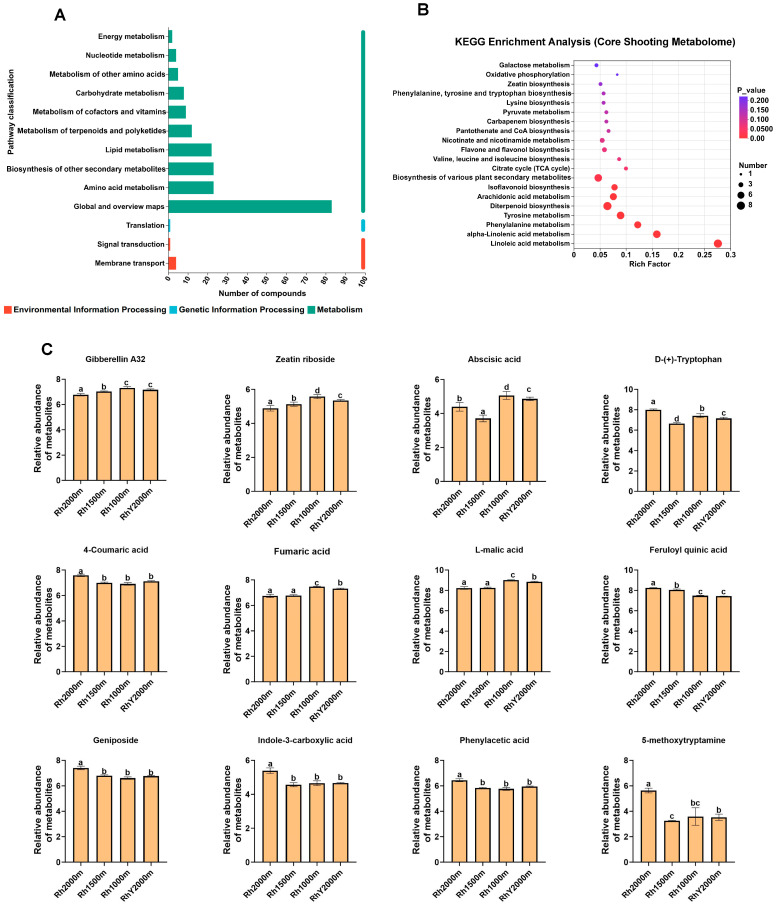
KEGG functional pathway statistics and enrichment analysis of the CSM metabolite set, and differential expression levels of selected metabolites. (**A**) Functional pathway statistics of the CSM metabolite set. (**B**) Functional pathway enrichment analysis of the CSM metabolite set. (**C**) Expression level analysis of differential metabolites, including hormones and signaling molecules (gibberellin A32, abscisic acid, zeatin riboside, D-(+)-tryptophan, indole-3-carboxylic acid, geniposide, phenylacetic acid, and 5-methoxytryptamine), energy metabolism-related substances (TCA cycle and carbohydrate metabolism metabolites: fumaric acid and L-malic acid), and cell wall remodeling and phenylpropanoid pathway-related metabolites (4-coumaric acid and feruloyl quinic acid). Relative abundance was calculated as the TIC-normalized peak area ratio of each metabolite to its mean value across all samples. Error bars represent standard deviations derived from five biological replicates. Different lowercase letters indicate significant differences (*p* < 0.05).

To gain functional insight into the CSM, we performed KEGG pathway mapping and enrichment analysis. The CSM encompassed a broad spectrum of metabolic categories, including energy, carbohydrate, amino acid, lipid, terpenoid, and secondary metabolism (Figure 4A). Enrichment analysis revealed that the CSM was significantly overrepresented in pathways central to shoot emergence: the tricarboxylic acid (TCA) cycle, oxidative phosphorylation, and pyruvate metabolism for energy provision; diterpenoid and zeatin biosynthesis for gibberellin and cytokinin synthesis; phenylalanine, tyrosine, and tryptophan biosynthesis for auxin and phenylpropanoid precursors; phenylpropanoid, flavonoid, and isoflavonoid pathways for cell wall remodeling and antioxidant defense; and linoleic acid metabolism for lipid signaling (Figure 4B, *p* < 0.05). These findings demonstrate that the CSM effectively captures the core metabolic reprogramming required for rhizome shooting in *F. rufa*.

To validate the CSM expression patterns, we quantified the relative abundances of 12 representative metabolites across the four groups (Figure 4C). The shooting groups (Rh_1500m, Rh_1000m, and RhY_2000m) exhibited significantly higher levels of gibberellin A32 and zeatin riboside compared to the non-shooting group Rh_2000m, with metabolite abundances converging consistently across all shooting groups. Notably, the metabolite profiles of RhY_2000m (warm gully center at 2000 m) and Rh_1000m (low-elevation warm zone at 1000 m) were virtually indistinguishable, with no significant differences detected for any measured metabolite. The mid-elevation Rh_1500m generally displayed intermediate abundances, consistent with its moderate soil temperature. These results confirm that the CSM faithfully represents the core metabolic reprogramming underlying bamboo rhizome shooting, and that localized warming at high elevations can fully restore the metabolic state characteristic of low-elevation conditions, thereby overcoming the low-temperature constraint imposed by elevation.

### 2.5. Transcriptomic Profiling of F. rufa Rhizomes Across Different Altitudes

**Figure 5:**
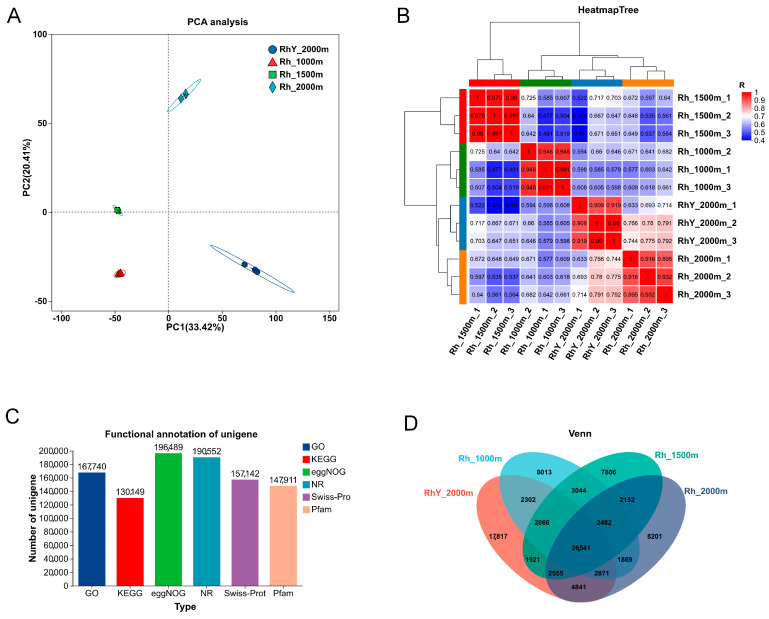
Transcriptome sequencing analysis of *F. rufa* rhizomes at different elevations and shooting statuses. (**A**) PCA of transcriptome sequencing samples from four *F. rufa* rhizome groups (Rh_1000m, Rh_1500m, RhY_2000m, and Rh_2000m). (**B**) Correlation analysis of transcriptome samples of *F. rufa* rhizomes. (**C**) Gene annotation statistics of all assembled genes and transcripts aligned against six major databases (NR, Swiss-Prot, Pfam, EggNOG, GO, and KEGG). (**D**) Venn analysis of *F. rufa* rhizome transcriptome samples.

To investigate the transcriptomic basis of elevation-dependent shooting in *F. rufa*, we performed RNA-seq analysis on rhizomes collected from four groups: Rh_1000m (shooting), Rh_1500m (shooting), RhY_2000m (shooting), and Rh_2000m (non-shooting) (Supplementary Table S3). Each of the 12 libraries generated over 5.41 Gb of clean data with Q30 scores exceeding 95.63%. De novo assembly using Trinity produced 375,166 unigenes (530,488 transcripts) with a mean N50 of 851 bp. Clean reads from individual samples mapped to the Trinity-assembled reference at rates ranging from 71.39% to 86.48%. Functional annotation against NR, Swiss-Prot, Pfam, EggNOG, GO, and KEGG databases yielded 369,695 annotated unigenes (Figure 5C), providing a solid reference for subsequent analyses. Sample correlation heatmap (Figure 5B) demonstrated high reproducibility among biological replicates within each group (all correlation coefficients > 0.96). PCA (Figure 5A) further resolved the clustering pattern: PC1 (33.42% variance) distinctly separated the non-shooting group Rh_2000m from all shooting groups (Rh_1000m, Rh_1500m, and RhY_2000m), while PC2 (20.41% variance) primarily reflected elevational differences. Notably, in contrast to the metabolomic PCA where RhY_2000m clustered closely with Rh_1000m, the transcriptomic PCA positioned RhY_2000m nearer to Rh_1500m and farther from Rh_1000m. This discrepancy implies that while warming at the high-elevation gully center drove metabolic convergence toward low-elevation conditions, the transcriptome may retain partial elevation-related “molecular memory” or be subject to additional regulation by non-thermal factors such as photoperiod and ultraviolet radiation.

Venn diagram analysis (Figure 5D) identified 26,541 genes expressed across all four groups (core transcriptome). Group-specific gene counts were 8013 (Rh_1000m), 7806 (Rh_1500m), 8201 (Rh_2000m), and 17,817 (RhY_2000m). The RhY_2000m group possessed approximately 2.2-fold more specific genes than the other three groups, which exhibited comparable numbers (7800–8200). This substantial transcriptional uniqueness of the high-elevation gully microenvironment suggests that, in addition to temperature-induced shooting, this habitat activates numerous distinct transcriptional programs, potentially in response to composite stresses including intense light exposure, large diurnal temperature fluctuations, and ultraviolet radiation. By contrast, shooting along the pure elevational gradient (Rh_1000m and Rh_1500m) and the non-shooting state (Rh_2000m) required relatively lower transcriptional specialization. These findings underscore the high transcriptional plasticity of *F. rufa* rhizomes in adapting to complex microenvironmental conditions.

### 2.6. Transcriptome-Wide Differential Expression and Functional Enrichment of Rhizome Genes

**Figure 6:**
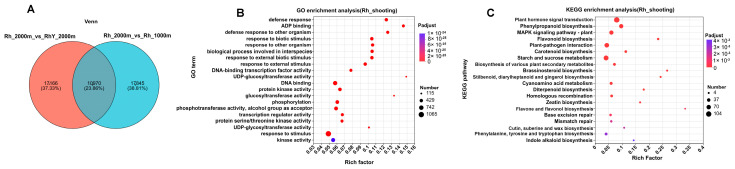
Differentially expressed gene analysis of *F. rufa* rhizomes across different elevations and shooting statuses. (**A**) Venn diagram analysis of differentially expressed gene (DEG) sets from different rhizome transcriptome comparisons (Rh_2000m vs. RhY_2000m and Rh_2000m vs. Rh_1000m). The two gene sets shared 10,970 differentially expressed genes, designated as the Rh_shooting gene set. (**B**) GO functional enrichment analysis of the Rh_shooting differentially expressed gene set. (**C**) KEGG functional pathway enrichment analysis of the Rh_shooting differentially expressed gene set.

To define the core gene set directly associated with rhizome shooting, we intersected differentially expressed genes (DEGs) from two independent comparisons, Rh_2000m vs. RhY_2000m (same elevation, 4 °C temperature difference, shooting vs. non-shooting) and Rh_2000m vs. Rh_1000m (elevational gradient, shooting vs. non-shooting), applying thresholds of |log_2_FC| ≥ 1 and padj < 0.05. Venn diagram analysis (Figure 6A) revealed 10,970 overlapping DEGs shared between the two contrasts. This common gene set, designated as Rh_shooting, represents the transcriptional signature most directly associated with the shooting phenotype, effectively excluding changes attributable solely to elevation or microenvironmental variation. GO functional enrichment analysis (Figure 6B; Supplementary Table S4) indicated that Rh_shooting genes were significantly overrepresented in biological processes including defense response, response to other organisms, response to biotic stimulus, and response to external stimulus, as well as in molecular functions such as DNA-binding transcription factor activity, protein kinase activity, UDP-glycosyltransferase activity, and phosphotransferase activity. These findings suggest that during shooting, bamboo rhizomes engage not only in transcriptional reprogramming and protein phosphorylation cascades but also in active responses to soil-borne pathogens or environmental stresses.

KEGG pathway enrichment analysis (Figure 6C; Supplementary Table S5) further revealed that Rh_shooting genes were predominantly involved in plant hormone signal transduction (gibberellin, auxin, abscisic acid, and brassinosteroid pathways), phenylpropanoid biosynthesis (lignin and flavonoids), MAPK signaling (plant-specific), flavonoid biosynthesis, starch and sucrose metabolism, diterpenoid biosynthesis (gibberellin), zeatin biosynthesis (cytokinin), plant–pathogen interactions, cutin/suberin/wax biosynthesis, and phenylalanine/tyrosine/tryptophan biosynthesis (auxin precursors). These results are highly consistent with the metabolomic CSM findings, further corroborating that temperature-induced shooting entails coordinated regulation of hormone signaling, energy metabolism, cell wall remodeling, defense responses, and antioxidant protection. The Rh_shooting gene set thus constitutes a valuable resource for constructing shooting regulatory networks and prioritizing candidate genes, including transcription factors, receptor kinases, and hormone biosynthetic enzymes.

### 2.7. Temporal Expression Patterns of Rh_Shooting Genes Across Temperature Gradients

**Figure 7:**
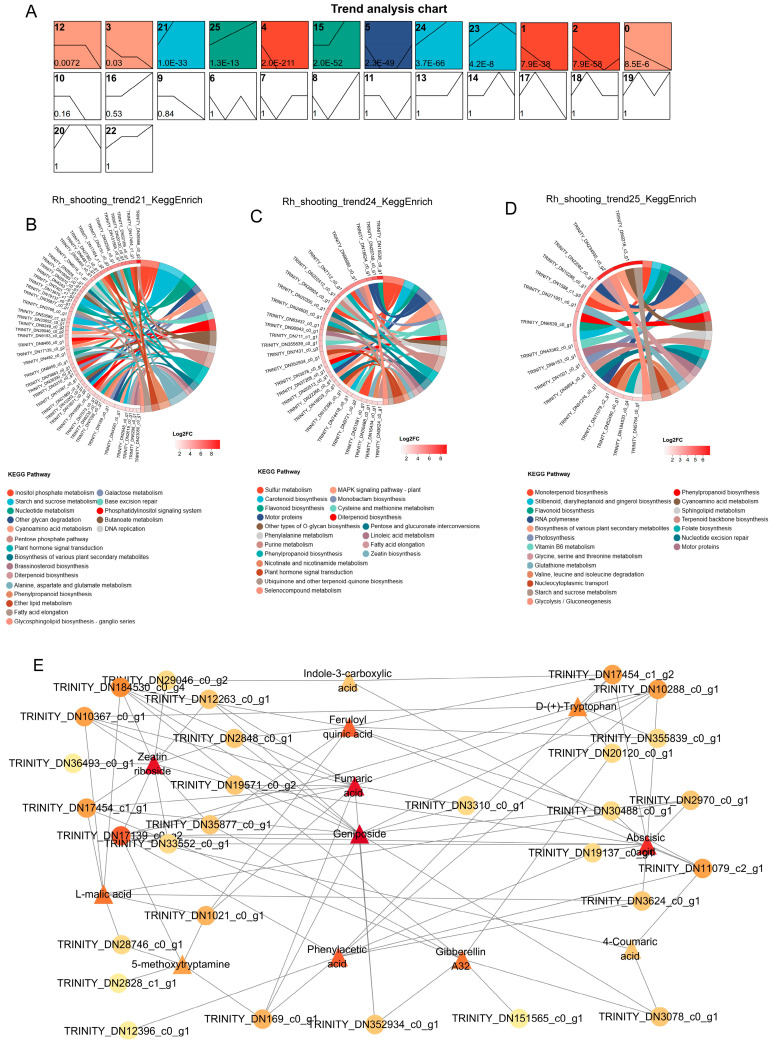
Temporal expression trends and integrated gene-metabolite network of the Rh_shooting gene set. (**A**) Significantly enriched temporal expression profiles from the STEM analysis of the Rh_shooting gene set across the four sample groups ordered by increasing soil temperature (Rh_2000m → Rh_1500m → Rh_1000m → RhY_2000m). Among the 26 significant trend modules (*p* < 0.05), three trends (21, 24, and 25) exhibited clear biological interpretability and close association with shooting regulation: profile 21 (shooting-on type, 511 genes, low-high-high-high), profile 24 (temperature-gradient type, 368 genes, low-medium-high-high), and profile 25 (microhabitat-enhanced type, 161 genes, low-medium-high-highest). (**B**–**D**) KEGG enrichment chord diagram of temporal expression of Trend 21, Trend 24, and Trend 25 gene sets, showing the top 20 enriched KEGG pathways and their constituent genes. The left side displays genes/transcripts arranged in descending order of log_2_FC; larger log_2_FC values indicate greater upregulation fold changes, smaller log_2_FC values indicate greater downregulation fold changes, and log_2_FC values approaching zero indicate smaller differential expression fold changes. The right side shows the KEGG pathway information significantly enriched with differentially expressed genes/transcripts. (**E**) Correlation network linking the 50 genes with 12 core shooting metabolites (CSM). Edges represent significant Pearson correlations (|r| > 0.8, *p* < 0.05); red = positive, blue = negative. Node colors: genes are coded by functional category (same as (**B**–**D**)), with darker colors indicate a greater number of associated genes and metabolites; metabolites are coded by chemical class, with triangles representing metabolites, and circles representing genes. The network reveals strong intra-module correlations within hormone, energy, cell wall, and defense modules, as well as trend-specific association patterns.

To characterize the dynamic expression behavior of the Rh_shooting gene set (10,970 DEGs) along the temperature gradient, we performed temporal trend analysis (STEM) across the four sample groups ordered by increasing soil temperature (Rh_2000m → Rh_1500m → Rh_1000m → RhY_2000m). This analysis resolved the genes into 26 significant trend modules (*p* < 0.05), among which three (Trends 21, 24, and 25) exhibited clear biological interpretability and close association with shooting regulation (Figure 7A; Supplementary Tables S6 and S8).

Trend 21 (“shooting-switch type,” 511 genes, *p* = 8 × 10^33^) comprised genes with low expression in Rh_2000m (non-shooting, cold) and uniformly high expression across all three shooting groups (Rh_1500m, Rh_1000m, and RhY_2000m), following a low–high–high–high pattern. These genes, acting as core shooting activators, are fully induced once soil temperature exceeds the ~20 °C threshold. KEGG enrichment revealed predominant involvement of plant hormone signal transduction, diterpenoid biosynthesis (gibberellin), brassinosteroid biosynthesis, phenylpropanoid biosynthesis, starch and sucrose metabolism, the pentose phosphate pathway, and the phosphatidylinositol signaling system, collectively functioning as molecular switches driving rhizome dormancy-to-germination transition (Supplementary Figure S4). Trend 24 (“temperature-dose type,” 368 genes, *p* = 3.7 × 10^−66^) displayed a progressive expression increase from Rh_2000m to Rh_1500m to Rh_1000m, peaking at Rh_1000m and maintaining comparable levels at RhY_2000m (low–medium–high–high). This pattern reflects a dose-dependent response to rising temperature. KEGG enrichment implicated these genes in plant hormone signal transduction, zeatin biosynthesis (cytokinin), diterpenoid biosynthesis, phenylpropanoid biosynthesis, flavonoid biosynthesis, carotenoid biosynthesis, the MAPK signaling pathway, linoleic acid metabolism, and phenylalanine metabolism, suggesting that post-initiation temperature elevation further amplifies hormone signaling, energy supply, and environmental adaptability. Trend 25 (“microenvironment-enhanced type,” 161 genes, *p* = 1.3 × 10^−13^) showed lowest expression in Rh_2000m, gradual increases through Rh_1500m and Rh_1000m, and maximal expression in RhY_2000m (low–medium–high–highest). Notably, despite similar soil temperatures between RhY_2000m and Rh_1000m, RhY_2000m exhibited significantly higher expression, indicating additional induction by non-thermal microenvironmental factors such as enhanced light intensity and UV radiation. KEGG enrichment highlighted flavonoid biosynthesis, phenylpropanoid biosynthesis, stilbenoid/diarylheptanoid/gingerol biosynthesis, photosynthesis, monoterpenoid biosynthesis, glutathione metabolism, glycolysis/gluconeogenesis, and starch and sucrose metabolism, functions that align with plant responses to intense light and UV stress, reflecting the unique transcriptomic imprint of the open high-elevation gully-center habitat. Among the remaining 23 significant trend modules, the majority exhibited expression patterns that were not directly interpretable in the context of shooting regulation. For instance, some trends showed highest expression specifically in Rh_2000m (non-shooting) or Rh_1500m (mid-elevation), while others displayed non-monotonic fluctuations across the temperature gradient (e.g., low–high–low–medium or high–low–medium–low). These modules were not prioritized for further analysis as their expression dynamics lacked consistent association with either temperature progression or shooting status.

### 2.8. Chord Diagram Visualization of Trend-Specific Pathway Enrichment

Based on the KEGG enrichment results for Trends 21, 24, and 25, we constructed chord diagrams to visualize the distribution of trend-specific genes across the top 20 significantly enriched pathways (Figure 7B–D; expression heatmaps for all genes within each trend are provided in Supplementary Figure S5). In these diagrams, the left semicircle represents the three trend gene sets, the right semicircle denotes enriched pathways, connecting line width reflects the number of enriched genes from each trend in a given pathway, and node size is proportional to log_2_FC. The three trends exhibited markedly distinct pathway preferences: Trend 21 was primarily anchored to core shooting pathways (plant hormone signal transduction, phenylpropanoid biosynthesis, and starch and sucrose metabolism; Figure 7B); Trend 24 was significantly enriched in pathways related to environmental signal perception and adaptive metabolism (MAPK signaling, flavonoid biosynthesis, carotenoid biosynthesis, zeatin biosynthesis, and linoleic acid metabolism; Figure 7C); and Trend 25 was concentrated in light-response and antioxidant defense pathways (flavonoid biosynthesis, photosynthesis, monoterpenoid biosynthesis, stilbenoid/diarylheptanoid/gingerol biosynthesis, and glutathione metabolism; Figure 7D).

Specifically, the highest log_2_FC gene in Trend 21 (*TRINITY_DN39366_c0_g2*, log_2_FC = 9.63) mapped to the pentose phosphate pathway, while multiple key pathways—plant hormone signal transduction (log_2_FC range 1.73–5.51), phenylpropanoid biosynthesis (1.51–4.86), and starch and sucrose metabolism (1.46–2.96)—were each covered by multiple highly expressed genes, reflecting coordinated multi-pathway activation during shooting initiation. Several genes participated in multiple pathways simultaneously (e.g., *TRINITY_DN169_c0_g1* is involved in degradation of other glycans, galactose metabolism, and glycosphingolipid biosynthesis), indicating their hub roles in the core shooting regulatory network. In Trend 24, the top gene (*TRINITY_DN15538_c0_g1*) was enriched in flavonoid biosynthesis, and MAPK signaling and plant hormone signal transduction shared multiple highly expressed genes (e.g., *TRINITY_DN12396_c0_g1* and *TRINITY_DN352934_c0_g1*), suggesting MAPK-mediated integration of environmental signals into the hormone signaling network. Enrichment of zeatin biosynthesis and carotenoid biosynthesis reflected positive regulation of cytokinin and ABA precursor synthesis by elevated temperature. In Trend 25, the top gene (*TRINITY_DN9216_c3_g1*) simultaneously participated in biosynthesis of various secondary metabolites, cyanoamino acid metabolism, and starch and sucrose metabolism, reflecting multi-pathway cross-response driven by the gully microenvironment. The presence of photosynthesis-related genes suggested that rhizome tissues may possess light-responsive or photosynthetic capacity under strong light, while co-enrichment of flavonoid biosynthesis, glutathione metabolism, and stilbenoid/diarylheptanoid/gingerol biosynthesis indicated that Trend 25 primarily functions in oxidative stress defense against intense light and UV radiation. Collectively, the chord diagrams reveal a functional continuum from core shooting initiation (Trend 21) through temperature-enhanced adaptation (Trend 24) to microenvironmental stress response (Trend 25), constituting the transcriptional regulatory network through which temperature and microenvironment synergistically drive rhizome shooting.

### 2.9. Gene–Metabolite Correlation Network Reveals Hub Regulators

To explore the regulatory relationships between genes and metabolites, we performed Pearson correlation analysis (|r| > 0.8, *p* < 0.05) between the genes from the top 20 enriched pathways and the 12 core shooting metabolites (CSM): gibberellin A32, abscisic acid, zeatin riboside, D-(+)-tryptophan, 4-coumaric acid, fumaric acid, L-malic acid, feruloyl quinic acid, geniposide, indole-3-carboxylic acid, phenylacetic acid, and 5-methoxytryptamine. The resulting network (Figure 7E) comprised 90 gene nodes and 12 metabolite nodes, with all edges representing significant positive correlations (*p* < 0.05) and correlation coefficients ranging from 0.80 to 0.96. Zeatin riboside occupied the most central position in the network, exhibiting significant positive correlations with the largest number of genes (e.g., *TRINITY_DN17139_c0_g2*, *TRINITY_DN184530_c0_g4*, *TRINITY_DN10367_c0_g1*, and *TRINITY_DN12263_c0_g1*) and forming a tightly connected module with gibberellin A32, fumaric acid, D-(+)-tryptophan, indole-3-carboxylic acid, and geniposide. This central position indicates that cytokinin signaling serves as a hub in the shooting regulatory network, potentially exerting global coordinating functions through interactions with other hormone and energy metabolism pathways. In contrast, abscisic acid displayed a distinctly different association pattern; its connected genes (e.g., *TRINITY_DN17139_c0_g2*, *TRINITY_DN33552_c0_g1*, *TRINITY_DN11079_c2_g1*, and *TRINITY_DN2970_c0_g1*) were all downregulated in shooting groups, consistent with ABA’s established role as a germination inhibitor.

From the gene-node perspective, *TRINITY_DN17139_c0_g2*, *TRINITY_DN184530_c0_g4*, *TRINITY_DN10367_c0_g1*, and *TRINITY_DN12263_c0_g1* occupied key hub positions, each significantly positively correlated with more than four core metabolites, suggesting that the enzymes or regulatory factors they encode may coordinate multiple metabolic branches. Secondary hubs included *TRINITY_DN17454_c1_g2* (associated with D-(+)-tryptophan, feruloyl quinic acid, phenylacetic acid, abscisic acid, and indole-3-carboxylic acid), *TRINITY_DN10288_c0_g1* (associated with geniposide, D-(+)-tryptophan, abscisic acid, phenylacetic acid, and fumaric acid), *TRINITY_DN1021_c0_g1* (associated with 5-methoxytryptamine, feruloyl quinic acid, L-malic acid, and fumaric acid), and *TRINITY_DN2970_c0_g1* (associated with geniposide, abscisic acid, and L-malic acid), which served as bridging connections between metabolic modules. Notably, network genes could be traced to specific trend categories: hub genes from Trend 21, such as *TRINITY_DN17139_c0_g2*, were primarily anchored to hormone signaling and cell wall metabolites (zeatin riboside, 4-coumaric acid, geniposide); hub genes from Trend 24, such as *TRINITY_DN184530_c0_g4*, were extensively associated with energy metabolites (fumaric acid, L-malic acid) and hormone metabolites (zeatin riboside, gibberellin A32, feruloyl quinic acid); and hub genes from Trend 25, such as *TRINITY_DN1021_c0_g1*, were distinctly associated with photosynthesis/defense-related metabolites (5-methoxytryptamine, L-malic acid, feruloyl quinic acid). This modular structure quantitatively validates, at the molecular interaction level, the coordinated regulatory mechanism of cytokinin/gibberellin signal activation, energy metabolism enhancement, cell wall remodeling, and defense metabolism reinforcement during temperature-induced shooting, and reveals the additional shaping effect of the high-elevation gully microenvironment through unique gene–metabolite association modules.

### 2.10. qRT-PCR Validation and Integrated Regulatory Model

**Figure 8:**
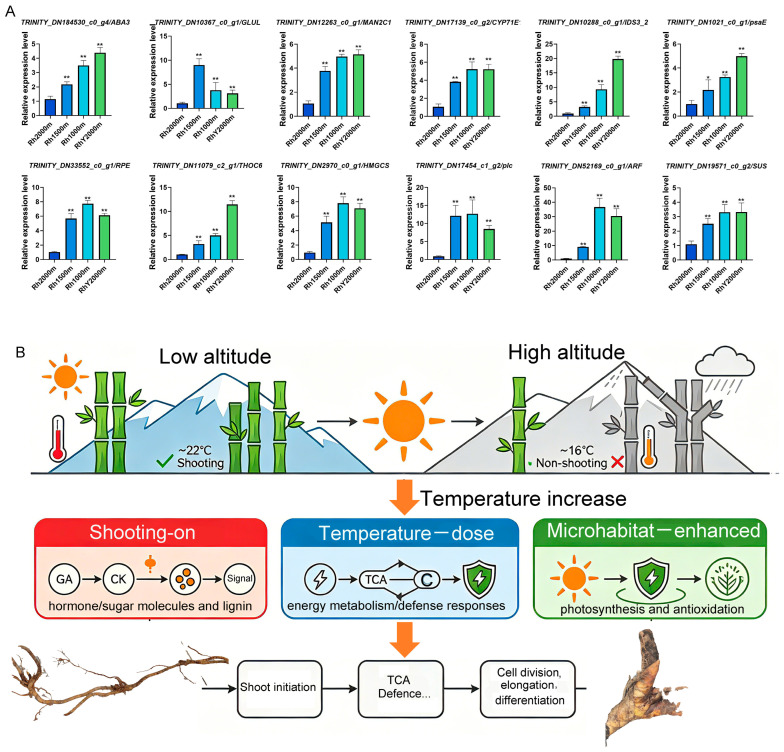
Validation and integrative model of temperature- and microhabitat-driven rhizome shooting in *F. rufa*. (**A**) qRT-PCR validation of six selected genes from the Rh_shooting set across the four sample groups. The asterisk represents the significance analysis (* *p* < 0.05 and ** *p* < 0.05). (**B**) A working model integrating altitude, microhabitat, temperature, and the molecular networks.

To validate the transcriptome data and consolidate the core findings, we performed qRT-PCR on 12 key genes selected from the Rh_shooting set based on their high log_2_FC values, high connectivity in the gene–metabolite network, coverage of all three trend clusters (Trends 21, 24, and 25), and representation of the four core functional pathways (hormone signaling, energy metabolism, cell wall remodeling, and defense response). The qRT-PCR results (Figure 8A) confirmed that the expression patterns of all six representative genes were highly consistent with the RNA-seq data.

Based on the multi-omics evidence, we constructed a four-tier cascade model of “environmental signal → signal transduction → molecular network → phenotypic output” (Figure 8B). Temperature decreases along the elevational gradient (1000 m: 22 °C → 1500 m: 20 °C → 2000 m gully edge: 16 °C), but the high-elevation gully center receives strong solar radiation, resulting in local warming (+4 °C, reaching 20.5 °C) that reverses the low-temperature inhibition imposed by elevation. Temperature and light signals activate the MAPK cascade and downstream transcription factors (ARF/MYB), converting environmental signals into molecular instructions. The three trend categories cooperate through functional division: Trend 21 (shooting-switch type) dominates hormone signaling (GA/cytokinin), cell wall remodeling (phenylpropanoid), and carbohydrate metabolism (starch → sucrose), which are synchronously activated once temperature exceeds the 20 °C threshold; Trend 24 (temperature-dose type) enhances energy metabolism (TCA cycle → fumaric acid/malic acid) and defense responses (MAPK/plant–pathogen interaction), which intensify with increasing temperature; and Trend 25 (microenvironment-enhanced type) specifically enhances photosynthesis, flavonoid antioxidant metabolism, and glutathione metabolism in response to strong light/UV stress in the high-elevation gully. These molecular events collectively drive cell division and elongation, ultimately achieving bamboo shoot emergence. This model provides a molecular explanation for the natural pattern of “shooting increasing from low to high elevations, but occurring only in locally warm areas at high elevations.”

## 3. Discussion

The core finding of this study is that soil temperature, rather than elevation itself, is the primary environmental factor driving rhizome shooting in *F. rufa*. By sampling along an elevation gradient (1000 m, 1500 m, 2000 m) within the same river gully and establishing a critical paired comparison between the cold gully edge (16.4 °C, non-shooting) and warm gully center (21.1 °C, shooting, ΔT ≈ 4 °C) at 2000 m, we were able to disentangle the confounding effects of elevation from the main effect of temperature. This experimental design revealed a clear pattern: all sites with soil temperatures exceeding approximately 20 °C—including low-elevation Rh_1000m, mid-elevation Rh_1500m, and high-elevation gully-center RhY_2000m—underwent shooting, whereas sites below this threshold (high-elevation gully-edge Rh_2000m) remained dormant [20]. This finding is consistent with studies on temperate bamboo shooting phenology, which indicate that soil temperature must reach a critical threshold to trigger shoot germination; it also echoes research on *Neosinocalamus affinis*, which showed that daily shoot growth increment is closely correlated with temperature [27,28,29]. Notably, although the high-elevation gully-center site (RhY_2000m) differed by 1000 m in elevation from the low-elevation site (Rh_1000m), their similar soil temperatures (21.1 °C vs. 21.6 °C) resulted in convergent metabolomic and transcriptomic profiles with the low-elevation shooting group. This “metabolic convergence” phenomenon molecularly confirms that microenvironmental warming can completely reverse the low-temperature inhibition imposed by elevation. This result also explains why field observations of *F. rufa* shooting exhibit a “low-to-high elevation advancing” pattern—essentially a spatiotemporal reflection of temperature variation along the elevation gradient, rather than an effect of elevation per se. At higher altitudes, locally warmed microhabitats—such as sun-exposed gully centers—effectively offset the low-temperature constraint, thereby permitting shooting at high elevations while accounting for its confinement to specific warm microsites. Furthermore, in this study, soil total nitrogen, total phosphorus, total potassium, and pH did not show any consistent patterns correlated with shooting status across sites, further reinforcing the conclusion that temperature serves as the dominant factor. We acknowledge that moist soils (60–70% across all sites) may harbor diverse soil bacteria that could influence plant metabolites. However, soil moisture was consistent among sites, and our RDA confirmed that temperature—rather than moisture or other edaphic factors—was the dominant driver of metabolomic variation (Supplementary Figure S2). Moreover, the two 2000 m sites, which share similar soil conditions and moisture but differ by 4 °C in temperature, exhibited the most pronounced metabolic divergence, further supporting temperature as the primary factor. We agree that future integration of rhizosphere microbiome analysis would be valuable to dissect plant–soil–microbe interactions during shooting.

Integrated multi-omics analysis of metabolome and transcriptome enabled us to systematically delineate the molecular network through which temperature regulates rhizome shooting. We defined the core shooting metabolome (CSM) comprising 843 metabolites that consistently accumulated under all shooting conditions, which was characterized by gibberellin precursor gibberellin A32, auxin precursor D-(+)-tryptophan, TCA cycle intermediates (fumaric acid, malic acid), and phenylpropanoid compounds (4-coumaric acid, feruloyl quinic acid) [30]. Correspondingly, the core shooting transcriptome (Rh_shooting) comprised 10,970 genes, which temporal trend analysis resolved into three functionally distinct clusters: Trend 21 (“shooting switch type”), which was synchronously activated upon temperature threshold breakthrough and enriched in plant hormone signal transduction, phenylpropanoid biosynthesis, and starch/sucrose metabolism; Trend 24 (“temperature dose type”), which displayed progressive upregulation with increasing temperature and was enriched in energy metabolism (TCA cycle, oxidative phosphorylation) and defense responses (MAPK signaling pathway); and Trend 25 (“microenvironment enhancement type”), which was specifically upregulated in the high-elevation gully-center warm zone and was enriched in photosynthesis, flavonoid biosynthesis, and glutathione metabolism. This refined molecular differentiation pattern is highly consistent with known regulatory mechanisms of bamboo rhizome bud germination: in *Cephalostachyum pingbianense*, the transition from dormancy to germination is coordinately regulated by multiple pathways including carbohydrate metabolism, hormone signal transduction, cell wall biogenesis, and temperature response [31,32]; In *Phyllostachys edulis*, rhizome lateral bud development is likewise controlled by the coordinated interplay of meristem development, sugar metabolism, and plant hormone signaling [19]. Beyond the individual contributions of each trend cluster, a deeper examination of their functional interconnections reveals that these three modules are not operating independently, but rather form an integrated regulatory network through coordinated carbon partitioning, energy supply, and ROS signaling. Specifically, Trend 21 (“shooting switch type”) serves as the primary driver: upon activation of GA/cytokinin signaling following temperature threshold crossing, it upregulates starch degradation enzymes (AMY3) and sucrose synthase (SUS1), promoting the conversion of stored starch to sucrose [16,20]. This sucrose pool not only provides carbon skeletons and osmotic support for rapidly dividing shoot bud cells, but also serves as the primary substrate for Trend 24-associated energy metabolism [33]. The TCA cycle intermediates (fumarate and malate) accumulated through Trend 24 activation not only fuel ATP production via oxidative phosphorylation to meet the high energy demands of shoot emergence, but also participate in feedback regulation of hormone signaling pathways [16]—for instance, malate has been shown to modulate stomatal movement and influence auxin transport, while fumarate accumulation may affect the cellular redox state, indirectly modulating the activity of redox-sensitive transcription factors. Meanwhile, Trend 25 (“microenvironment enhancement type”)—which is specifically induced by strong light and UV radiation in the high-elevation gully center—upregulates flavonoid and glutathione metabolism, which scavenges reactive oxygen species (ROS) generated under high light/UV stress. This antioxidant defense not only protects actively dividing shoot tissues from photo-oxidative damage but also intersects with MAPK signaling, as ROS are well-established secondary messengers that modulate MAPK cascades, potentially providing a feedback loop that fine-tunes hormone signaling outputs under varying light conditions [34]. The coordinated interplay among the three trends—carbon flow from Trend 21 fueling Trend 24 energy metabolism, and ROS signaling from Trend 25 modulating Trend 21/24 outputs—illustrates that the shooting program is not a linear cascade but rather a tripartite synergistic network in which temperature signals are amplified, buffered, and integrated through metabolic and redox crosstalk to ensure robust shoot emergence under diverse environmental conditions.

Our integrated network analysis further revealed that zeatin riboside occupies a central position in the network, exhibiting significant positive correlations with the largest number of genes and forming tightly connected modules with core metabolites including gibberellin A32, fumaric acid, and tryptophan, indicating that cytokinin signaling plays a global coordinating role in the shooting regulatory network [20]. Multiple hub genes (e.g., *TRINITY_DN17139_c0_g2*, *TRINITY_DN184530_c0_g4*) were simultaneously associated with more than four core metabolites, suggesting that the enzymes or regulatory factors encoded by these genes may participate in the coordinated regulation of multiple metabolic branches. Although direct functional validation of these candidate regulators remains technically challenging in bamboo due to the lack of an established stable transformation system, future studies should pursue such validation once bamboo genetic transformation technologies mature. Regarding the upstream temperature sensor in rhizome tissue, although it remains to be identified, cyclic nucleotide-gated calcium channels (CNGCs) have been shown to function as plasma membrane thermosensors in land plants, translating temperature-induced changes in membrane fluidity into calcium signals that activate downstream MAPK cascades and heat shock responses [35,36]. Furthermore, single-cell or spatial transcriptomics could resolve whether the shooting-switch program originates in meristematic bud cells or surrounding storage tissue—a question that bulk RNA-seq cannot address [37]. The transcriptome–metabolome decoupling between RhY_2000m and Rh_1000m likely reflects a combination of factors: post-transcriptional regulation, differential turnover rates, persistent non-thermal factors (photoperiod and UV), and elevation-associated epigenetic memory—a mechanism that enables plants to retain transcriptional footprints of prior environmental conditions that are not reversed by short-term temperature changes [38]. 

Interestingly, although abscisic acid (ABA) is generally regarded as a germination inhibitor, its abundance was highest in Rh_1000m and RhY_2000m—two shooting sites exposed to strong light—whereas it was only at moderate levels in the non-shooting Rh_2000m. We hypothesize that when temperature conditions are no longer limiting for shoot initiation, the accumulated ABA in these sites may not function primarily to suppress germination, but rather could serves as a photoprotective signal to help rhizomes cope with oxidative stress induced by strong solar radiation in open low-elevation environments or high-elevation gully centers. The enrichment of the Trend 25 gene set (flavonoid biosynthesis, glutathione metabolism, and photosynthesis-related pathways) provides correlative support for this interpretation. Nevertheless, this observation is consistent with the notion that under suitable temperatures, gibberellin/cytokinin signaling can still dominate the shooting process, while ABA may adopt a more adaptive role in high-light environments [39,40]. The orderly functional differentiation of the three trend gene categories—”initiation → enhancement → microenvironmental specialization”—collectively constitutes the complete transcriptional regulatory network through which temperature and microenvironment synergistically drive bamboo rhizome shooting.

The findings of this study carry important ecological and conservation implications. First, bamboo shoots represent the highest nutritional value food source for giant pandas during spring and summer, with high protein and soluble sugar contents but low fiber content; the spatiotemporal dynamics of rhizome shooting directly determine giant panda movement patterns, habitat utilization, and population fitness [7,8]. Our molecular data demonstrate that local warm microhabitats in high-elevation gully centers can reverse elevation-imposed low-temperature limitations through approximately 4 °C warming, restoring shooting capability in high-elevation rhizomes—providing a mechanistic explanation at the molecular level for the “shoot-chasing” phenomenon (i.e., the foraging strategy of giant pandas tracking the “wave” of bamboo shoots from low to high elevations seasonally) [10]. Second, this study reveals the high transcriptional plasticity of *F. rufa* rhizomes in response to complex microenvironments: the number of specifically expressed genes at the high-elevation gully-center site (RhY_2000m; 17,817) was approximately 2.2-fold higher than in the other three groups (7800–8200), suggesting that this microenvironment not only induced shooting through temperature elevation but also activated numerous unique transcriptional programs through composite environmental stresses including intense light and UV radiation. This plasticity may represent a key adaptive strategy for bamboo to cope with variable environmental conditions along elevation gradients [12,26]. Finally, under the context of climate change, global warming may gradually drive soil temperatures in high-elevation cold zones toward or beyond the approximate 20 °C shooting threshold, thereby expanding the shootable range of *F. rufa* and increasing food resource availability for giant pandas in extreme habitats [41]. However, long-term warming may also bring potential risks such as soil moisture deficit, nutrient depletion, or phenological mismatch. Therefore, future research should combine long-term field monitoring with controlled-environment experiments to systematically assess the comprehensive effects of climate warming on *F. rufa* shooting dynamics and its coupling with giant panda foraging behavior, providing more robust scientific evidence for giant panda habitat conservation and food resource management.

## 4. Materials and Methods

### 4.1. Plant Materials

Plant materials were obtained from *F. rufa* populations growing in the Tangjiahe National Nature Reserve, Qingchuan County, Sichuan Province, China. Sampling was conducted along an elevational transect following a major gully, with three sites established at 1000 m, 1500 m, and 2000 m above sea level (a.s.l.), where ambient temperatures at the time of collection were 26 °C, 23.2 °C, and 20.8 °C, respectively. Because the shooting period of *F. rufa* is mainly in August and September, the collection campaign took place in mid-August 2025, during which the regional monthly mean air temperature was 20 °C, soil temperature at 10 cm depth averaged 17 °C, and soil moisture content was approximately 65%. The study area is characterized by a mean annual temperature of 16 °C, average relative humidity of 72%, an annual frost-free period of 243 days on average, and mean annual precipitation of 1021.7 mm.

At each elevation, rhizome tissues were harvested from five spatially independent bamboo clumps to serve as biological replicates, with a minimum separation distance of 10 m between adjacent sampling points to ensure genetic independence. Rhizome samples were collected by excavating 10–20 cm outward from the culm base of each selected clump, avoiding re-sampling from the same ramet. In total, 15 samples were obtained (five biological replicates, with additional samples for 2000 m microhabitat contrasts as needed), providing comprehensive coverage of the natural intraspecific variation within the study population. Immediately following collection, all samples were snap-frozen in liquid nitrogen and subsequently stored at −80 °C until nucleic acid extraction and metabolite profiling.

### 4.2. RNA Extraction and Quantitative Real-Time PCR (qRT-PCR) Validation

Total RNA was extracted from *F. rufa* rhizome tissues using TRIzol^®^ Reagent (Invitrogen, Carlsbad, CA, USA) strictly following the supplier’s protocol, encompassing sample homogenization, chloroform-mediated phase separation, isopropanol-based RNA precipitation, ethanol washing, and final dissolution in nuclease-free water. RNA quality and concentration were evaluated via agarose gel electrophoresis and spectrophotometric measurement (NanoDrop 2000, Thermo Scientific, Wilmington, DE, USA), ensuring integrity prior to reverse transcription. First-strand cDNA was synthesized from 1 µg of total RNA using the PrimeScript™ RT Reagent Kit with gDNA Eraser (Takara Bio, Shiga, Japan), incorporating a genomic DNA elimination step to exclude potential DNA contamination. Quantitative real-time PCR (qRT-PCR) was carried out on an Applied Biosystems 7500 Fast Real-Time PCR System (Thermo Fisher Scientific, Waltham, MA, USA) using TB Green™ Premix Ex Taq™ II (Takara Bio). The PCR regime consisted of an initial denaturation at 95 °C for 30 s, followed by 40 amplification cycles of 95 °C for 5 s and 60 °C for 34 s; a melting curve analysis was subsequently performed to confirm the specificity of each amplicon. The transcript encoding ubiquitin (*UBQ*, *TRINITY_DN1489_c0_g1*) was selected as the endogenous reference on the basis of its stable expression across all experimental groups in preliminary stability tests. Target-specific primer pairs were designed using Primer 5.0 software (Premier Biosoft, Palo Alto, CA, USA), yielding amplicons of 80–200 bp with melting temperatures adjusted to 58–62 °C; complete primer sequences are compiled in Supplementary Table S3. All qRT-PCR reactions were conducted in triplicate technical repeats, with three independent biological replicates processed per sample. Relative transcript levels were computed via the 2^−ΔΔCt^ method [42], normalizing to *UBQ* and calibrating to the sample with minimal expression. Amplicon specificity was further verified by agarose gel electrophoresis of the PCR end-products.

### 4.3. RNA Sequencing and Transcriptomic Data Processing

Total RNA was isolated from *F. rufa* rhizomes originating from three elevational sites (1000 m, 1500 m, and 2000 m a.s.l.) using the RNAprep Pure Plant Kit (Tiangen, Beijing, China), with three biological replicates per altitude. RNA quantity and purity were examined on a NanoDrop 2000 spectrophotometer (Thermo Scientific, Wilmington, DE, USA), while integrity was inspected via 1% agarose gel electrophoresis and RQN (RNA Quality Number) was determined using an Agilent 5300 Fragment Analyzer (Agilent Technologies, Santa Clara, CA, USA). Samples meeting all of the following specifications were advanced to library construction: total RNA ≥ 1 µg, concentration ≥ 30 ng µL^−1^, RQN > 6.5, and A_260_/A_280_ ratio within 1.8–2.2. Strand-specific cDNA libraries were generated and sequenced on an Illumina NovaSeq 6000 platform by Majorbio Bio-Pharm Technology Co., Ltd. (Shanghai, China).

Raw reads underwent stringent quality control to eliminate adapter contamination, poly-N tracts, and low-quality bases. Specifically, 3′-terminal bases with Phred scores < 20 were clipped; reads with any remaining base below Q10 or containing > 10% ambiguous nucleotides were discarded; and sequences shorter than 20 bp after trimming were excluded. Base-composition and quality profiles were monitored cycle-by-cycle to ensure data integrity. Clean reads from all groups were pooled for de novo transcriptome assembly using Trinity (version 2.8.5) [43], which employs three independent modules for processing large-scale RNA-seq data; the resulting contigs were subsequently refined and validated to produce the final transcript set. For functional characterization, all assembled unigenes were queried against six public repositories—NR, Swiss-Prot, Pfam, COG, GO, and KEGG—via BLASTX (E-value cut-off 8 × 10^5^) and HMMER (for Pfam), and annotation outcomes were compiled per database. Transcript abundances were estimated as TPM (transcripts per million) using RSEM (version 1.3.1) [44]. Differential expression analysis between pairwise comparisons was performed with both DESeq2 (version 1.30.1; |log_2_FC| ≥ 1, FDR < 0.05) [27] and DEGseq (|log_2_FC| ≥ 1, FDR < 0.001) [43], retaining only genes satisfying both criteria as robust DEGs. To interpret the functional implications of these DEGs, GO enrichment was executed via the Goatools package and KEGG pathway enrichment via Python **3.12**’s SciPy library, applying a Bonferroni-corrected significance threshold of *p* < 0.05 to identify overrepresented terms and pathways relative to the whole transcriptome background.

### 4.4. Metabolite Extraction and UPLC-MS/MS Profiling

*F. rufa* rhizomes were pulverized under liquid nitrogen, and the resulting powders were extracted using appropriate solvent systems for metabolomic analysis. Specifically, approximately 100 mg of frozen powder was weighed into a 2 mL centrifuge tube, and 1 mL of pre-chilled extraction solvent (methanol/water, 80:20, *v*/*v*) was added. The mixture was vortexed for 30 s and then sonicated in an ice-water bath for 10 min, followed by centrifugation at 12,000 rpm for 15 min at 4 °C. The supernatant was carefully transferred to a new tube, and the extraction was repeated once with an additional 0.5 mL of extraction solvent. The combined supernatants were evaporated to dryness under a gentle nitrogen stream. The dried residue was reconstituted in 200 µL of 50% methanol (*v*/*v*), vortexed for 30 s, sonicated for 5 min, and centrifuged at 12,000 rpm for 10 min at 4 °C. The final supernatant was filtered through a 0.22 µm PTFE membrane prior to UPLC-MS/MS analysis. Metabolic profiling was carried out on an ultra-performance liquid chromatography-tandem mass spectrometry (UPLC-MS/MS) platform, with metabolite identification based on fragmentation patterns from MS/MS spectra. The LC-MS system comprised a Thermo Scientific UHPLC-Q Exactive HF-X mass spectrometer (Thermo Fisher Scientific, Waltham, MA, USA) coupled to an ultra-high-performance liquid chromatograph. Chromatographic separation was achieved using an ACQUITY UPLC BEH C18 column (100 mm × 2.1 mm i.d., 1.7 µm; Waters, Milford, MA, USA) maintained at 40 °C. The mobile phase consisted of (A) water containing 0.1% formic acid and 2% acetonitrile, and (B) acetonitrile with 0.1% formic acid. Each sample was injected at a volume of 1 µL. Electrospray ionization (ESI) was employed, and mass spectral data were acquired in both positive and negative ion modes. For relative quantification, the peak area of each metabolite was normalized to the total ion current (TIC) of the corresponding sample. The relative abundance of each metabolite in a given sample was then calculated as the ratio of its normalized peak area to the mean normalized peak area of that metabolite across all samples. Raw MS data were subjected to principal component analysis (PCA) and sample correlation heatmaps to assess inter-sample reproducibility and overall variation. Differential metabolites were screened using orthogonal partial least squares discriminant analysis (OPLS-DA), with selection criteria set at variable importance in projection (VIP) > 1, *p* < 0.05, and absolute fold change (|FC|) ≥ 2, following established practices [45]. For functional interpretation, all differentially accumulated metabolites were mapped to the KEGG database to identify enriched biological pathways associated with the observed metabolic shifts.

### 4.5. Physiological and Biochemical Soil Analyses

Physicochemical properties of the soils surrounding the *F. rufa* rhizosphere samples were determined by Chengdu Baihui Biotechnology Co., Ltd. (Chengdu, China). Prior to analysis, all soil specimens were air-dried and passed through a 100-mesh sieve. Soil organic matter was quantified via the potassium dichromate oxidation-external heating method. Total nitrogen was determined by the Kjeldahl method following sulfuric acid-catalyst digestion. Available nitrogen was measured using the alkaline hydrolysis-diffusion procedure combined with Kjeldahl determination. Total phosphorus was assessed by the molybdenum-antimony colorimetric method after acid digestion, while available phosphorus was extracted with sodium bicarbonate or ammonium fluoride-hydrochloric acid and likewise determined by molybdenum-antimony colorimetry. Total potassium and available potassium were analyzed by flame photometry. Soil pH and temperature were recorded in situ using a portable pH meter and a soil thermometer, respectively.

### 4.6. Statistical Analysis

For metabolomic assays, five biological replicates were utilized; all other experiments included three independent biological replicates. Data are expressed as mean ± standard error of the mean (SEM). Differences among multiple groups were evaluated using one-way analysis of variance (ANOVA), and when a significant effect was detected (*p* < 0.05), post hoc pairwise comparisons were performed with Tukey’s Honest Significant Difference (HSD) test. Statistical significance was defined as adjusted *p* < 0.05. All statistical computations were executed with GraphPad Prism 9.0 (GraphPad Software, San Diego, CA, USA).

## 5. Conclusions

In summary, our integrated multi-omics analysis establishes that soil temperature, rather than elevation per se, is the primary driver of rhizome shooting in *F. rufa*, with an apparent threshold near 20 °C, and local warming of ~4 °C in high-elevation microhabitats is sufficient to fully restore shooting competence. We define a core shooting metabolome of 843 metabolites and a core shooting transcriptome of 10,970 genes that resolve into three functionally distinct temporal clusters—shooting-on, temperature-dose, and microenvironment-enhanced—collectively orchestrating hormone signaling, energy metabolism, cell wall remodeling, and antioxidant defenses. These findings establish a molecular framework linking altitudinal temperature variation to bamboo shoot availability, providing mechanistic insights into giant panda foraging ecology and offering a basis for predicting climate change impacts on panda habitat quality.

## Data Availability

Data are contained within the article and Supplementary Materials.

## Supplementary Materials

The following supporting information can be downloaded at: https://www.mdpi.com/article/10.3390/plants15152302/s1, Figure S1: Overall phenotypic observation of rhizomes, culms, and bamboo shoots of Fargesia rufa at 1000 m (shoot emergence), 1500 m (shoot emergence), and 2000 m (shoot emergence and pre-emergence) elevations; Figure S2: Redundancy analysis (RDA/CCA) of soil factors with metabolites/gene expression; Figure S3: Circular pie chart analysis of primary and secondary metabolites in the metabolomic study; Figure S4: KEGG and GO functional enrichment analysis of temporal expression Trend 21, Trend 24, and Trend 25 gene sets; Figure S5: Gene expression heatmaps of temporal expression Trend 21, Trend 24, and Trend 25 gene sets; Table S1: Statistical summary of compound classifications from metabolomic analysis; Table S2: The set of 843 metabolites obtained by [(Rh_1000m vs. Rh_2000m) ∩ (RhY_2000m vs. Rh_2000m)] \ (Rh_1000m vs. RhY_2000m); Table S3: Statistical table of transcriptome sequencing data for each rhizome sample of *Fargesia rufa*; Table S4: GO enrichment analysis statistics of the Rh_Shooting differentially expressed gene set; Table S5: KEGG functional pathway enrichment analysis statistics of the Rh_Shooting differentially expressed gene set; Table S6: Detailed table of kegg pathway enrichment analysis for gene expression trend 21, 24 and 25; Table S7: Gene expression matrix (TPM) of temporal expression Trend 21, Trend 24, and Trend 25 gene sets; Table S8: Time series expression trend analysis result table; Table S9: Primers for key genes in the study.
