# Supplementary material for: Comparative Multi-Omics Analysis of Rhizome Shooting in Fargesia rufa Under Altitudinal Temperature Variation
Source: Plants (Basel). 2026 Jul 27;15(15):2302. doi: 10.3390/plants15152302 (PMC13468535; doi:10.3390/plants15152302)
Supplement: Supplementary file 1 [file plants-15-02302-s001.zip › Supplementary files.pdf]

Comparative Multi-Omics Analysis of Rhizome Shooting in *Fargesia rufa* Under Altitudinal Temperature Variation

Xin Zhao [1,2,3,†], Man Tang [1,†], Yanwen Zhao [1], Mengqiu Chen [1], Xiaojun Wang [4], Qi Lin [4], Zhijian Long [1,2,3,]*
and Shanglian Hu [1,2,3,]*

[1] College of Life Science and Agri-Forestry, Southwest University of Science and Technology,
Mianyang 621000, China
[2] Sichuan Provincial Forestry and Grass Land Key Laboratory for Conservation and Sustainable Utilization of Bamboo Genetic Resources in Southwest of China, Mianyang 621010, China
[3] Key Laboratory of Bamboo Cultivation and Management, National Forestry and Grassland Administration, Mianyang 621000, China
[4] The Conservation of Endangered Wildlife Key Laboratory of Sichuan Province, Sichuan Academy of Giant Panda, Chengdu 610000, China
* Correspondence: long20053182@swust.edu.cn (Z.L.); hushanglian@swust.edu.cn (S.H.)
[†] These authors contributed equally to this work.

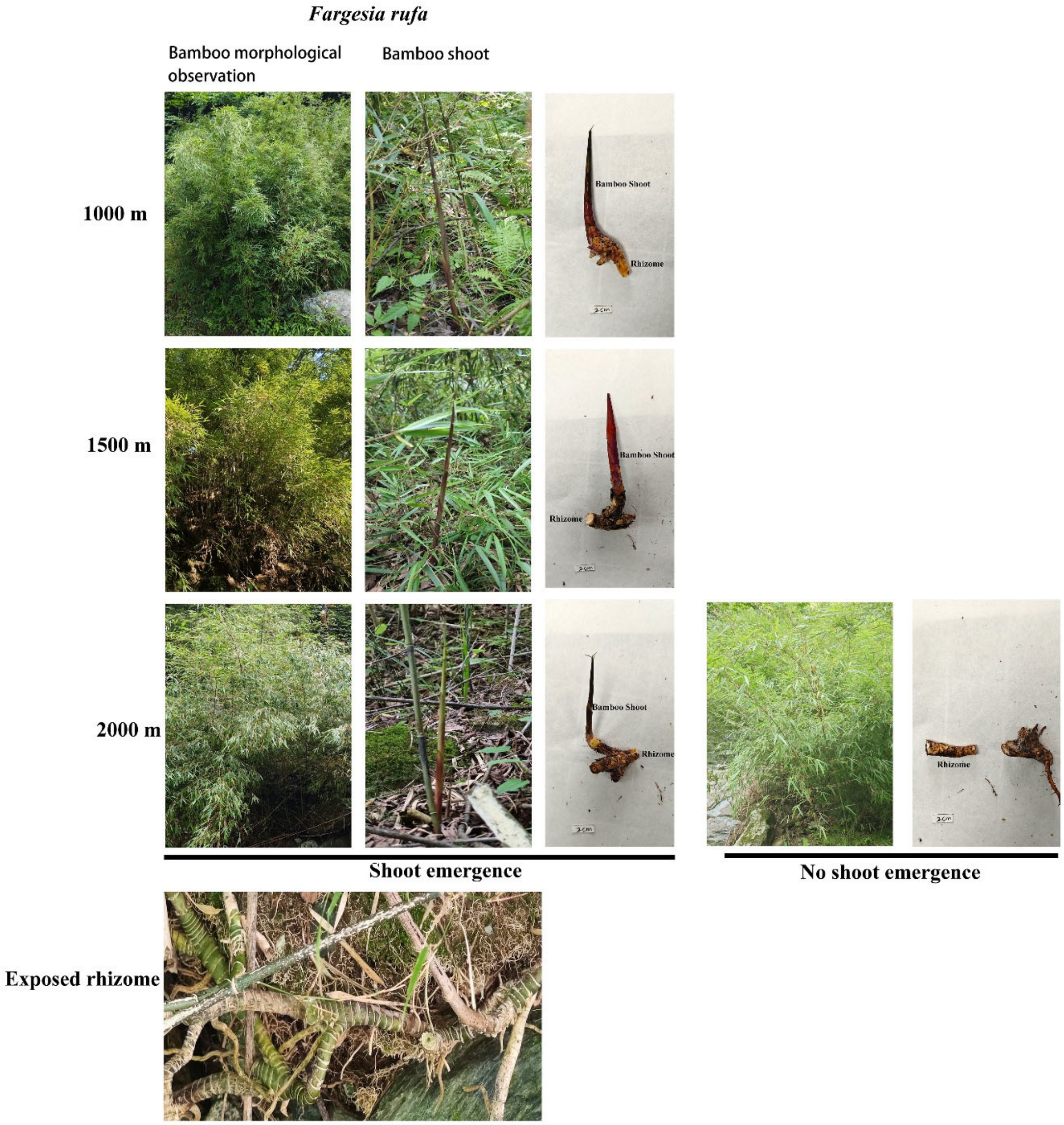

Supplementary figure S1. Overall phenotypic observation of rhizomes, culms, and bamboo shoots of *Fargesia rufa* at 1000 m (shoot emergence), 1500 m (shoot emergence), and 2000 m (shoot emergence and pre-emergence) elevations.

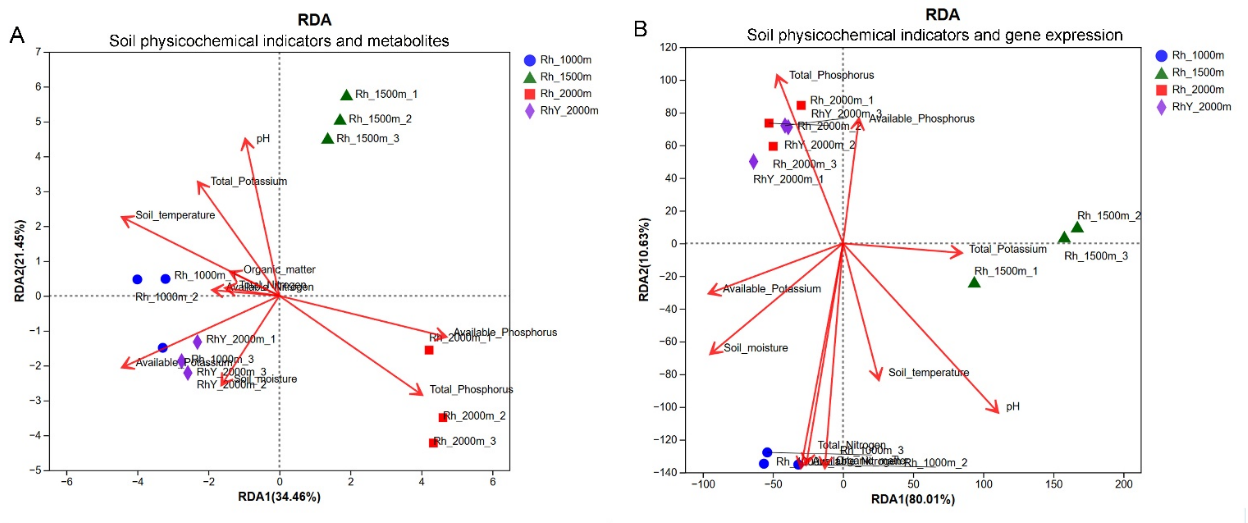

Supplementary Figure S2. Redundancy analysis (RDA/CCA) of soil factors with metabolites/gene expression.

(A) RDA/CCA plot of soil factors and metabolites. (B) RDA/CCA plot of soil factors and gene expression. In the figure, points of different colors or shapes represent sample groups from different environments or conditions; species are represented by green arrows by default in RDA plots; species are represented by green inverted triangles in CCA plots; red arrows indicate quantitative environmental factors, with the length of environmental factor arrows representing the magnitude of their explanatory power (degree of influence) on species data; the angle between environmental factor arrows represents positive or negative correlations (acute angle: positive correlation; obtuse angle: negative correlation; right angle: no correlation); projecting sample points onto the arrows of quantitative environmental factors, the distance from the projection point to the origin represents the relative magnitude of the environmental factor's influence on the distribution of the sample community.

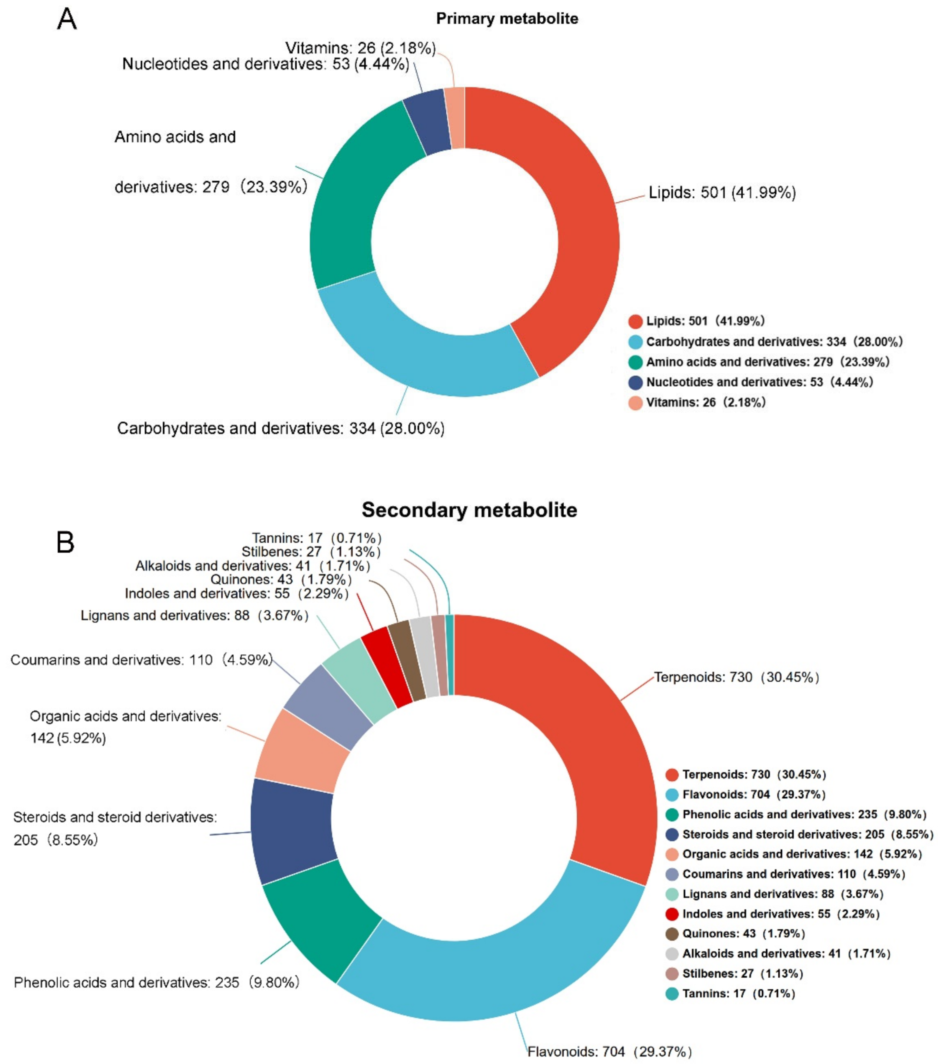

Supplementary figure S3. Circular pie chart analysis of primary and secondary metabolites in the metabolomic study.

Note: Based on the selected classification (plant compound classification, plant primary metabolite classification, plant secondary metabolite classification), metabolites are displayed in descending order of abundance within each category, showing the number of metabolites and their respective percentages. Different colors in each pie chart represent distinct classifications, with the sector area indicating the relative proportion of metabolites in that category.

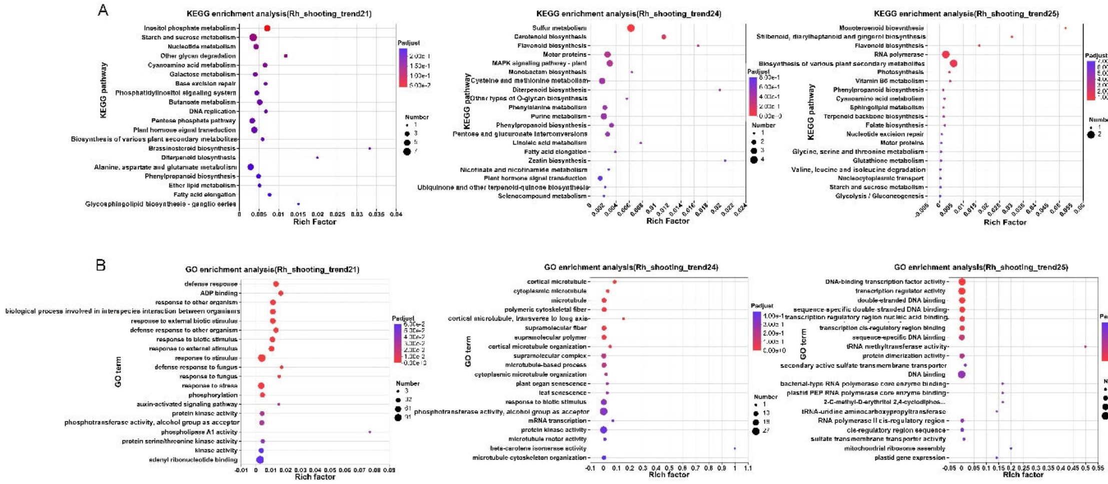

Supplementary figure S4. KEGG and GO functional enrichment analysis of temporal expression Trend 21, Trend 24, and Trend 25 gene sets.

(A) KEGG functional pathway enrichment analysis of temporal expression Trend 21, Trend 24, and Trend 25 gene sets. (B) GO functional enrichment analysis of temporal expression Trend 21, Trend 24, and Trend 25 gene sets.

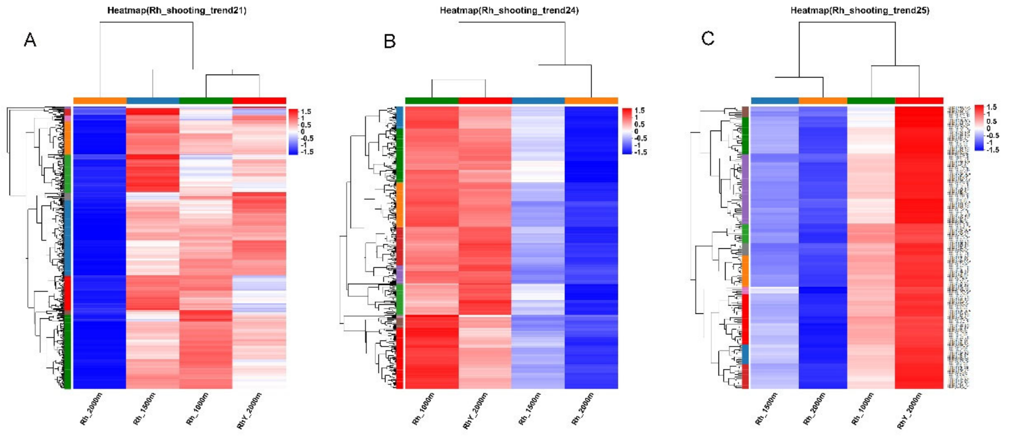

Supplementary figure S5. Gene expression heatmaps of temporal expression Trend 21, Trend 24, and Trend 25 gene sets.
